# Optimal Niacin Requirement of Oriental River Prawn *Macrobrachium nipponense* as Determined by Growth, Energy Sensing, and Glycolipid Metabolism

**DOI:** 10.1155/2022/8596427

**Published:** 2022-09-10

**Authors:** Jing-Wen Wang, Yi-Cheng Che, Miao Sun, Yi-Qing Guo, Bo Liu, Xiang-Fei Li

**Affiliations:** ^1^Key Laboratory of Aquatic Nutrition and Feed Science of Jiangsu Province, College of Animal Science and Technology, Nanjing Agricultural University, No. 1 Weigang Road, Nanjing 210095, China; ^2^Key Laboratory of Freshwater Fisheries and Germplasm Resources Utilization, Ministry of Agricultural and Rural Affairs, Freshwater Fisheries Research Center, Chinese Academy of Fishery Sciences, Wuxi 214081, China

## Abstract

Niacin is indispensable for the growth and development of aquatic animals. However, the correlations between dietary niacin supplementations and the intermediary metabolism of crustaceans are still poorly elucidated. This study explored the effects of different dietary niacin levels on the growth, feed utilization, energy sensing, and glycolipid metabolism of oriental river prawn *Macrobrachium nipponense*. Prawns were fed with different experimental diets containing graded niacin levels (15.75, 37.62, 56.62, 97.78, 176.32, and 339.28 mg/kg, respectively) for 8 weeks. Weight gain, protein efficiency, feed intake, and hepatopancreas niacin contents all maximized in the 176.32 mg/kg group with significance noted with the control group (*P* <0.05), whereas the opposite was true for feed conversion ratio. Hepatopancreas niacin concentrations increased significantly (*P* < 0.05) as dietary niacin levels increased, and peaked at the 339.28 mg/kg group. Hemolymph glucose, total cholesterol, and triglyceride concentrations all maximized in the 37.62 mg/kg group, while total protein concentration reached the highest value in the 176.32 mg/kg group. The hepatopancreas mRNA expression of AMP-activated protein kinase *α* and sirtuin 1 peaked at the 97.78 and 56.62 mg/kg group, respectively, and then both decreased as dietary niacin levels increased furtherly (*P* < 0.05). Hepatopancreas transcriptions of the genes related to glucose transportation, glycolysis, glycogenesis, and lipogenesis all increased with increasing niacin levels up to 176.32 mg/kg, but decreased significantly (*P* < 0.05) as dietary niacin levels increased furtherly. However, the transcriptions of the genes related to gluconeogenesis and fatty acid *β*-oxidation all decreased significantly (*P* < 0.05) as dietary niacin levels increased. Collectively, the optimum dietary niacin demand of oriental river prawn is 168.01-169.08 mg/kg. In addition, appropriate doses of niacin promoted the energy-sensing capability and glycolipid metabolism of this species.

## 1. Introduction

Niacin, also called nicotinic acid, vitamin B_3_, and the anti-leprosy factor, is an indispensable water-soluble vitamin for animals [1]. Being an important component of coenzyme I (NAD^+^) and II (NADP^+^), niacin participates in more than 300 redox reactions in vivo [2], especially in the transfer of both H^+^ and electron in the redox metabolism of carbohydrates, lipids, and proteins, thus playing crucial roles in energy metabolism and biosynthesis pathway [3]. Indeed, it has been reported that niacin can regulate the lipid metabolism of animals through various hormones and regulatory factors like the G protein-coupled receptor, diacylglycerol acyltransferase, silent information regulator 1 (SIRT1), and adenosine monophosphate-activated protein kinase (AMPK) [4]. In addition, niacin can govern the glucose metabolism by targeting SIRT1 and AMPK, which can consequently mediate the pathways of glycolysis, glycogenesis, gluconeogenesis, and so on [5]. However, unlike the case in mammals, aquatic animals lack the ability to convert tryptophan to niacin [6], thus often exhibiting niacin deficiency symptoms like anorexia, poor feed utilization, growth retardation, and increased mortality when fed niacin-deficient diets [7]. To date, the optimal niacin requirement has been evaluated in many fish species, like 28 mg/kg for carp *Cyprinus carpio* [8], 26 mg/kg for tilapia *Oreochromis mossambicus* [9], 7.4 mg/kg for channel catfish *Ictalurus punctatus* [10, 11], 33 mg/kg for rohu *Labeo rohita* and mrigal *Cirrhinus mrigala* [12], 30.62-31.25 mg/kg for blunt snout bream *Megalobrama amblycephala* [13], and 63-83 mg/kg for gilthead seabream *Sparus aurata* [14]. Unlikely, the optimal niacin requirement has been investigated only in several shrimp and prawn species, like 7.2 mg/kg for grass shrimp *Penaeus monodon* [15], 109.6 mg/kg for Pacific white shrimp *Litopenaeus vannamei* [16], 250 mg/kg for Indian white prawn *Penaeus indicus* [1], and 400 mg/kg for kuruma prawn *Penaeus japonicus* [17]. However, previous literatures generally focused on growth and feed utilization. The correlations between dietary niacin supplementation and the intermediary metabolism of aquatic species are still poorly elucidated.

The intermediary metabolism is controlled by a complex network composed of various nutrient sensors, among which AMPK*α* and SIRT1 have both attracted considerable attention [18]. The AMPK*α*-SIRT1 pathway is widely acknowledged as an energy sensing network, which governs the cellular energy balance [19]. Once activated, AMPK*α* could depress hepatic gluconeogenesis and lipogenesis, while stimulate the uptake of glucose and the *β*-oxidation of fatty acids, thus leading to the enhanced energy consumption [20]. These metabolic adjustments are also facilitated by SIRT1, since it and AMPK*α* can activate each other to form a positive feedback loop that precisely regulates the metabolic homeostasis [18]. Until now, the correlations between niacin and the energy sensing and glucose/lipid metabolism of aquatic animals are still poorly elucidated. To date, only one literature is accessible indicating that dietary nicotinamide supplementation benefits the glycolipid metabolism and glucose homeostasis of blunt snout bream offered a carbohydrate-enriched diet by mediating SIRT1 and its coactivators [21]. Nevertheless, the potential roles of niacin in the energy sensing and intermediary metabolism of crustaceans are still barely understood.

As an economically crucial freshwater prawn species, oriental river prawn *Macrobrachium nipponense* has been widely cultured in China. Due to the high protein and amino acid content and delicious flavor, aquaculture of this species has gained considerable attention in the east region of China [22]. Currently, the culture cost of this species is relatively high mainly due to the lack of high-efficiency artificial feed. This underscores the urgency of investigating the nutrients' demand of this species. Nowadays, the optimal requirements of macro-nutrients have been well characterized in oriental river prawn [23–28]. However, the vitamin requirement of this species has still been barely evaluated, which brings great difficulties to the development of nutritionally balanced diets for this species. Considering the important roles of niacin in energy metabolism, this study aimed to evaluate the optimal niacin requirements of oriental river prawn in terms of growth performance and feed utilization. Besides, the correlations between niacin doses and energy sensing and glucose/lipid metabolism were also elucidated. The present results could facilitate the development of high-efficiency diets for this species, as could lower the farming cost.

## 2. Materials and Methods

### 2.1. Experimental Diets

Firstly, a semi-purified basal diet was prepared using casein, gelatin, and fish meal as protein sources, fish oil and soybean oil as lipid sources, and corn starch as the sole carbohydrate source. The premix contained all the vitamins and minerals required by prawn except for niacin. Then, a total of 6 experimental diets (Table 1) were prepared by supplementing graded doses of niacin (0, 20, 40, 80, 160, and 320 mg/kg) to the basal diet, respectively. The actual dietary niacin concentrations were 15.75, 37.62, 56.62, 97.78, 176.32, and 339.28 mg/kg diet, respectively, quantified by the high-performance liquid chromatography (HPLC) method [29].

All raw materials were crushed through a 60-mm mesh, and were weighted according to the feed formula. Then, all the protein sources were mixed thoroughly with corn starch. The premix and niacin were both mixed by the gradual enlargement method to improve the efficiency of mixing. Other powder ingredients were then added for further mixing. After that, both soybean oil and fish oil were supplemented for a thorough mix. Last a certain proportion (15% of feed weight) of water was supplemented to turn the feed powder into dough, which was then pelleted by a double screw extrusion machine. The feed was then broken into a particle size of 1.0 mm, dried, and stored at -20°C for subsequent use.

### 2.2. Feeding Experiment

Healthy *M. nipponense* (Taihu No.1) were purchased from an experimental hatch farm near Tai Lake (Jiangsu province, China). Prawns were adapted to the culture conditions for 2 weeks by feeding the control diet in several indoor tanks. After that, 720 prawns (average weight: 0.18 g) were collected and divided into 24 indoor tanks (30 prawns per tank) randomly with 4 replicates for each group. Prawns were fed at 8:00, 12:00, and 16:00 h each day for 8 weeks. After 2 h of feeding, a 45.5 cm long siphon drain was used to clear the uneaten feed and feces with the feed intake recorded. Dead prawns were also collected and weighted. During the feeding trial, several artificial water grasses were placed in the tanks for shielding. The water temperature and dissolved oxygen were maintained at 26.0 ± 0.5°C and above 5 mg/L, respectively. In addition, 1/3 of water was replaced every two days to meet the basic demand of prawns for water quality.

### 2.3. Sampling

Prawns were subjected to a 24 h fast to empty the intestinal content when the feeding trial terminated. Then, the number and weight of prawns within each tank were recorded. Six prawns were randomly selected from each tank for hemolymph and hepatopancreas. Briefly, 1 mL aseptic syringe was used to extract hemolymph from the pericardial sinus of the posterior edge of the breastplate. Then, samples were placed into Eppendorf tubes and subjected to a centrifugation (3,500 rpm at 4°C for 10 min) with the supernatant kept in the refrigerator at -80°C. Thereafter, the hepatopancreas was separated and subjected to liquid nitrogen frozen followed by preservation at -80°C.

### 2.4. Analytic Protocols

#### 2.4.1. Growth Performance Determination

The calculation formulas of the growth performance parameters were presented as follows:

(1) $$\begin{array}{c} \text{Weight gain}\left(\text{WG},\%\right)=\left[\left(W_{2}-W_{1}\right)/W_{1}\right]*100,\\ \text{Specific growth rate }\left(\text{SGR},\%/\text{d}\right)=\left[\left(\ln\;W_{2}-\ln W_{1}\right)/T\right]*100,\\ \text{Survival ratio}\left(\text{SR},\%\right)=N_{t}/N_{0}*100,\\ \text{Feed conversion ratio}\left(\text{FCR}\right)=W_{3}/\left(W_{2}-W_{1}\right),\\ \text{Protein efficiency ratio}\left(\text{PER}\right)=\left(W_{2}-W_{1}\right)/\left(W_{3}*\text{CP}\right)*100, \end{array}$$

where *W*_1_ and *W*_2_ is the initial and final body weight, respectively, *W*_3_ is total feed intake, *N*_*t*_ is final prawn number, *N*_0_ is initial prawn number, *T* is the culturing days, and CP is the protein content in the diets.

#### 2.4.2. Determination of Proximate Composition and Niacin Contents

The proximate composition of the experimental diets was determined by the following methods: moisture by oven drying at 105°C for a constant weight; crude protein (N ×6.25) by the Kjeldahl System; crude lipid by ether extraction; and ash by combustion in a muffle furnace (550°C for 5 h).

The HPLC method was adopted to quantify the hepatopancreas and dietary niacin contents [29]. In short, approximately 0.5 g of hepatopancreas or diets was homogenized with hydrochloric acid (3 mL, 0.01 mol/L) followed by the protein precipitation using trichloroacetic acid (1 mL, 10%). The mixture was then subjected to a 10,260 rpm centrifugation for 10 min at 4°C. The supernatant was purified with ether for three times. Then, the ether was vaporized, followed by a filtration using a 0.45 *μ*m membrane. The supernatant was then analyzed by a HPLC device (HP1100, America). The sample was derivatized with a mixture consisted of potassium ferricyanide (0.1%) and sodium hydroxide (15%). The mobile phase was performed using a mixture containing potassium hydrogen phosphate (25 mmol/L) and methanol (v:v, 85 : 15) under a 1.0 mL/min flow rate. Then, a fluorescence detector (excitation wave length: 360 nm, emission wave length: 425 nm) was used to quantify the niacin content.

#### 2.4.3. Hemolymph Metabolites Detection

Hemolymph total protein (TP), glucose (GLU), triglyceride (TG), and total cholesterol (TC) concentrations were all quantified by the OLYMPUSAU600 biochemical analyzer using the bicinchoninic acid assay [30], the glucose oxidase method [31], and the glycerol-3-phosphate oxidase p-aminophenol method (for TG) and the cholesterol oxidase-peroxidase coupling method (for TC) [32], respectively.

#### 2.4.4. Transcriptional Analysis

Real-time fluorescence quantitative polymerase chain reaction (PCR) was adopted to determine the relative transcriptions of the genes involved in energy sensing and glycolipid metabolism in the hepatopancreas of prawns. Briefly, the total RNA of hepatopancreas was extracted by the Trizol reagent. After verifying its purity, the synthesis of cDNA was conducted using a Prime Script™ RT-PCR kit, and was amplified by a SYBR® Premix Ex Taq ™ II kit. Primers were designed according to the available cDNA sequences of this species from previous literatures and Genbank (Table 2), and were synthesized later. The reaction system (22.5 *μ*L) included 2 *μ*L of cDNA template, 10 *μ*L of 2 × QuantiNova SYBR® Green PCR Master mix, 0.5 *μ*L of upstream and downstream primers, respectively, and 9.5 *μ*L of ddH_2_O. Briefly, the PCR reaction conditions were followed by a total of 40 cycles: pre-denaturation at 95°C for 1 min, denaturation at 95°C for 10 s, and extension at 60°C for 15 s. Then, the 2^-*ΔΔ*Ct^ method [33] was adopted to determine the relative transcriptions of genes using *β-actin* as the internal reference [34].

### 2.5. Statistics

Data were tested for homogeneity (the Shapiro-Wilk test) and normality (Levene's test) before the conduction of one-way analysis of variance (ANOVA). Then, the means were ranked by Turkey's multiple range test if significance (*P* < 0.05) was noted. The orthogonal polynomial contrasts were also adopted to evaluate the types (namely, linear, quadratic, or cubic) of significance [35] with the data shown as means ± S.E.M. Statistical significance was set as *P* < 0.05, and the extreme significance was set as *P* < 0.001. In addition, the broken-line regression analysis (between weight gain and hepatopancreas niacin contents against dietary niacin levels, respectively) was also adopted to quantify the optimum niacin demand of oriental river prawn.

## 3. Results

### 3.1. Growth and Feed Utilization

**Figure 1:**
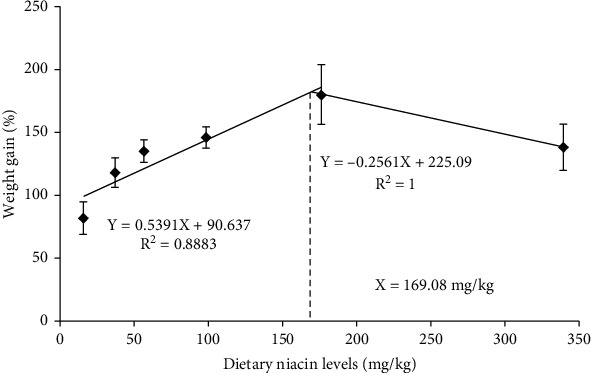
Relationship between dietary niacin levels (mg/kg) and weight gain (%) of oriental river prawn.

There was no significance noted in SR (*P* > 0.05) among all the treatments (Table 3). However, WG, SGR, feed intake (FI), FCR, and PER were all significantly (*P* < 0.05) affected in a linear pattern. Besides, FCR was also significantly (*P* < 0.05) affected in a quadratic pattern. Significantly (*P* < 0.05) high values of WG, SGR, FI, and PER were noted in the 176.32 mg/kg group compared with the control group, while the opposite was true for FCR. The optimum dietary niacin demand of oriental river prawn was 169.08 mg/kg (Figure 1) according to the broken-line regression analysis of WG against dietary niacin levels.

### 3.2. Hepatopancreas Niacin Contents and Hemolymph Metabolites Concentrations

**Figure 2:**
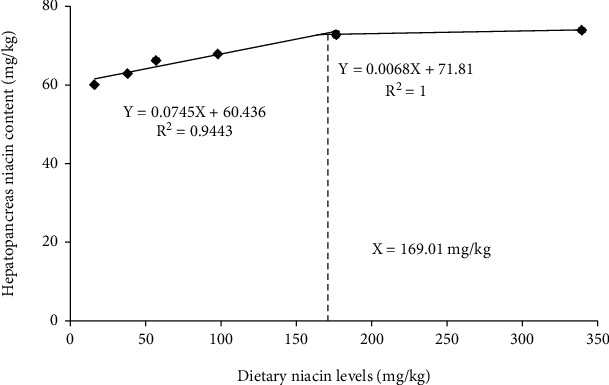
Relationship between dietary niacin levels (mg/kg) and hepatopancreas niacin contents (mg/kg) of oriental river prawn.

Hepatopancreas niacin content was significantly (*P* < 0.001) affected in a linear pattern (Table 4). It increased significantly (*P* < 0.05) with increasing niacin levels up to 176.32 mg/kg, then plateaued as niacin levels increased furtherly (*P* > 0.05). The optimum dietary niacin demand of oriental river prawn was 168.01 mg/kg (Figure 2) according to the broken-line regression analysis of hepatopancreas niacin contents against dietary niacin levels. Additionally, the GLU, TP, and TG concentrations were all significantly (*P* < 0.05) affected in a linear pattern, while a significant (*P* < 0.05) linear and cubic pattern was observed in the TC concentration. The GLU concentration of the 37.62 mg/kg group was significantly (*P* < 0.05) higher than those of the 176.32 and 339.28 mg/kg groups, but showed no statistical difference (*P* > 0.05) with those of the rest groups. The TG concentration of the 37.62 mg/kg group was significantly (*P* < 0.05) higher than those of the 56.62 and 339.28 mg/kg groups, but showed no statistical difference (*P* > 0.05) with those of the other groups. A significantly (*P* < 0.05) high value of TP concentration was noted in the 176.32 mg/kg group compared with the 15.75 mg/kg group. The TC concentration of the 37.62 mg/kg group was significantly (*P* < 0.05) higher than those of the other groups except for the 56.62 mg/kg group.

### 3.3. The Transcriptions of Energy Sensing-Related Genes

**Figure 3:**
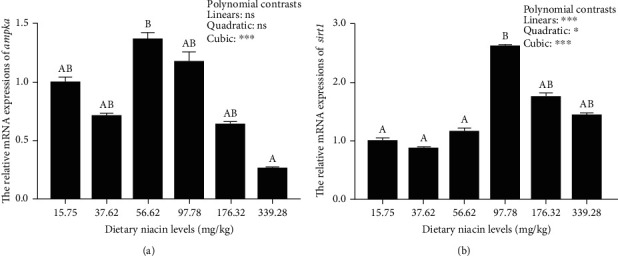
Relative transcriptions of the genes involved in energy sensing in the hepatopancreas of oriental river prawn fed diets differing in niacin levels. For tissue expression, data are referred to the values obtained in prawn fed the basal diet. Each data represents the mean of eight replicates (four individuals, each was tested twice as technical replicates). Bars assigned with different superscripts are significantly different (*P* < 0.05). ^∗^*P* < 0.05, ^∗∗∗^*P* < 0.001, ns: not significant. *ampkα*: AMP-activated protein kinase *α*; *sirt1*: sirtuin 1.

The transcription of *ampkα* was significantly (*P* < 0.001) affected in a cubic pattern, while a significant (*P* < 0.05) linear, quadratic, and cubic pattern was noted in that of *sirt1* (Figure 3). The *ampkα* transcription of the 56.62 mg/kg group was significantly (*P* < 0.05) higher than that of the 339.28 mg/kg group, but showed no statistical difference (*P* > 0.05) with those of the rest groups. The *sirt1* transcription of the 37.62 mg/kg group was significantly (*P* < 0.05) lower than that of the 97.78 mg/kg group, but showed no statistical difference (*P* > 0.05) with those of the other groups.

### 3.4. Relative Transcriptions of the Genes Related to Glycolipid Metabolism

**Figure 4:**
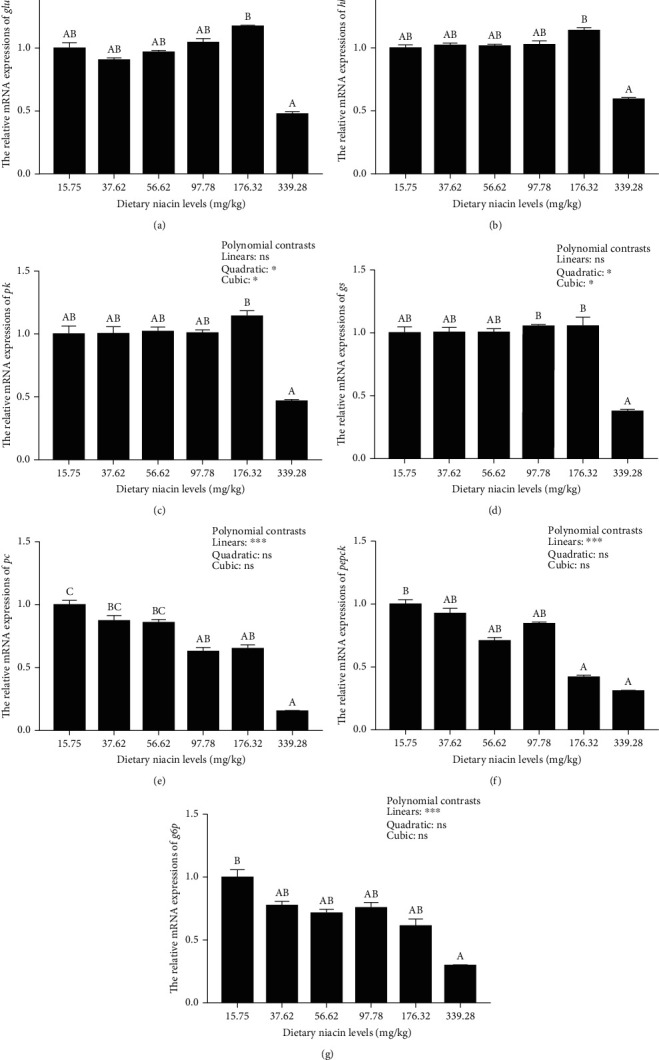
Relative transcriptions of the genes involved in glucose metabolism in the hepatopancreas of oriental river prawn fed diets differing in niacin levels. For tissue expression, data are referred to the values obtained in prawn fed the basal diet. Each data represents the mean of eight replicates (four in individuals, each was tested twice as technical replicates). Bars assigned with different superscripts are significantly different (*P* < 0.05). ^∗^*P* < 0.05, ^∗∗∗^*P* < 0.001, ns: not significant. *glut2*: glucose transporter 2; *hk*: hexokinase; *pk*: pyruvate kinase; *gs*: glycogen synthase; *pc*: pyruvate carboxylase; *pepck*: phosphoenolpyruvate carboxykinase; *g6p*: glucose-6-phosphatase.

**Figure 5:**
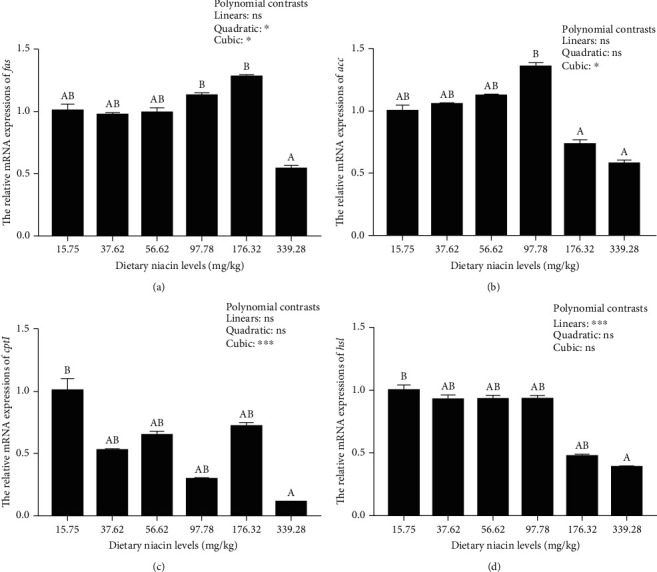
Relative transcriptions of the genes involved in lipid metabolism in the hepatopancreas of oriental river prawn fed diets differing in niacin levels. For tissue expression, data are referred to the values obtained in prawn fed the basal diet. Each data represents the mean of eight replicates (four individuals, each was tested twice as technical replicates). Bars assigned with different superscripts are significantly different (*P* < 0.05). ^∗^*P* < 0.05, ^∗∗∗^*P* < 0.001, ns: not significant. *fas*: fatty acid synthase; *acc*: acetyl-CoA carboxylase; *cptI*: carnitine palmitoyltransferase I; *hsl*: hormone-sensitive triglayceride lipase.

The transcriptions of *glut2*, *hk*, *pk*, *gs*, and *fas* were all significantly (*P* < 0.05) affected in a quadratic and cubic pattern, while a significant (*P* < 0.001) linear pattern was noted in those of *pc*, *pepck*, *g6p*, and *hsl* (Figures 4 and 5). Besides, the transcriptions of *acc* and *cptI* were both significantly (*P* < 0.05) affected in a cubic pattern. The hepatopancreas transcriptions of *glut2*, *hk*, *gs*, *pk*, and *fas* all increased with increasing niacin levels up to 176.32 mg/kg, but decreased significantly (*P* < 0.05) as dietary niacin levels increased furtherly. A similar result was also noted in the transcription of *acc*, which maximized in the 97.78 mg/kg group. However, the transcriptions of *pc*, *pepck*, *g6p*, *cptI*, and *hsl* all decreased significantly (*P* < 0.05) as dietary niacin levels increased.

## 4. Discussion

As a vital vitamin, niacin is crucial for the normal growth of aquatic animals. An inadequate intake of this nutrient inevitably induces a series of deficiency symptoms in aquatic organisms. The WG, SGR, and PER of oriental river prawn in this study all improved remarkably as dietary niacin levels increased, and all maximized in the 176.32 mg/kg group. However, the opposite was true for FCR. Supportively, similar results were also reported in other aquatic species like grass shrimp and Indian carps (namely, *C. mrigala* and *L. rohita*) [15, 36]. According to a previous literature, niacin is essential for the metabolism of macro-nutrients, and its supplementation at suitable doses could improve the feed utilization and growth performance of prawns [1]. The optimum niacin demand of oriental river prawn is 169.08 mg/kg as determined by the regression analysis of WG against dietary niacin levels. The higher niacin requirement of prawn, compared with most fish species, was justifiable. Generally, unlike fish, crustaceans eat slowly. The feed particles will be immersed in water for a long time before being eaten, thus resulting in the increased dissolution rate of water-soluble vitamins. In addition, the leaking of water-soluble vitamins would be further accelerated by the pellet-eating process of crustaceans [3]. This inevitably leads to the higher niacin demand of crustaceans compared with fish. A growth retardation coupled with the poor survival rate and feed efficiency of oriental river prawn was noted in the control group in this study, but no other obvious deficiency symptoms were noticed, as was similar to that reported in grass shrimp [15]. In addition, high doses of niacin (namely, 339.28 mg/kg) also resulted in the retarded growth of oriental river prawn coupled with the poor feed efficiency, although no significance was observed. Supportively, an excessive intake of niacin generally resulted in an inhibition of hematopoietic and immune functions in aquatic animals [13], as would consequently result in the poor growth performance of prawn. However, further validations are needed.

Currently, hepatopancreas niacin contents are commonly determined to evaluate the optimum niacin demand of aquatic species apart from growth performance and nutrients utilization [13, 37]. The hepatopancreas niacin contents of oriental river prawn in this study generally increased as dietary niacin levels increased from 15.75 to 176.32 mg/kg, but plateaued with furthering increasing niacin doses. This indicated a saturation of niacin deposition in the hepatopancreas of prawn offered a high dose of niacin (namely, 339.28 mg/kg). Supportively, supra-optimal dietary niacin levels also resulted in a saturated hepatopancreas niacin content in the Indian white prawn coupled with the retarded growth and low survival rate [1].

Niacin is also closely involved in the metabolism of macro-nutrients in aquatic animals [38]. Accordingly, the effects of niacin on the hemolymph metabolites concentrations of oriental river prawn were investigated in this study. The concentrations of glucose, protein, and total cholesterol all increased first then decreased with increasing dietary niacin levels, while that of triglyceride generally decreased. It has been demonstrated in human medicine that niacin has dual functions. For example, it can prevent pellagra at low doses (0.3 mg/kg per day), but could largely affect the lipid metabolism after a high intake (7-60 mg/kg per day) [39]. Similarly, diets supplemented with a certain amount of niacin also remarkably increased the serum total cholesterol content of adult Gift tilapia (*Oreochromis niloticus*), but decreased the triglyceride content [40]. Generally, niacin can be converted into nicotinoylglycine assisted by coenzyme A, thus preventing stem cells from using coenzyme A to synthesize cholesterol [41]. This would undoubtedly lead to the decreased cholesterol content with the increasing niacin supplementations. In addition, the increased glucose and protein concentrations might be indicative of an enhanced carbohydrate and protein utilization by oriental river prawn fed appropriate niacin levels. However, the underlying mechanisms are still unknown. Further studies investigating the effects of niacin on the digestive and absorptive functions of prawn are needed.

In the present study, dietary supplementation of niacin at 56.62 and 97.78 mg/kg up-regulated the transcriptions of *ampkα* and *sirt1*, respectively, suggesting that niacin could promote the energy sensing capability of oriental river prawn. Supportively, as an evolutionary conserved serine/threonine protein kinase, AMPK*α* governs the cellular energy homeostasis, and is generally regarded as a key energy sensor [42]. In addition, as a NAD^+^-dependent histone deacetylase, SIRT1 also plays important regulatory roles in energy metabolism [19]. Both AMPK*α* and SIRT1 can activate each other to form a positive feedback loop, thus governing intracellular energy metabolism [43]. Indeed, niacin supplementation has been demonstrated to increase the cellular production of NAD^+^, which can consequently activate SIRT1 in fish [21]. Then, SIRT1 can deacetylate liver kinase B1 (LKB1) at Lys48, thus triggering its activation. Being an upstream kinase, LKB1 can in turn activate AMPK*α* by phosphorylating Thr172 in the catalytic subunit of AMPK*α* [44], thus mediating intermediary metabolism. However, further studies at the translational and post-translational levels are needed to validate this.

Once activated, both AMPK*α* and SIRT1 could increase the energy expenditure by adjusting glycolipid metabolism, thus maintaining energy homeostasis [19]. In this study, high doses of niacin (176.32 mg/kg) remarkably up-regulated the expressions of *glut2*, *pk*, *hk*, and *gs* in the hepatopancreas of oriental river prawn at the transcriptional level, but decreased those of *pepck*, *pc*, and *g6p*. These results indicated that niacin could enhance the glucose transport, glycolysis, and glycogen synthesis of this species when supplemented at appropriate doses, but depress the gluconeogenesis pathway. It is widely acknowledged that (1) GLUT2 is a bidirectional glucose transporter, ensuring the movement of glucose into and out of hepatocytes [45]; (2) both PK and HK govern the glycolysis pathway, while PEPCK is the rate-limiting enzyme of the gluconeogenesis pathway, which also involves PC and G6Pase [46]; (3) and GS governs the glycogenesis pathway as a rate-limiting enzyme [47]. This was in line with the results noted in the hemolymph glucose concentrations of oriental river prawn in this study. In addition, appropriate doses of niacin also remarkably up-regulated the expressions of both *fas* and *acc* at the transcriptional level, but inhibited those of both *cptI* and *hsl*, suggesting that niacin supplementation could promote fatty acids synthesis in oriental river prawn, while inhibit lipid mobilization. Supportively, both ACC and FAS play crucial roles in the biosynthesis of fatty acids, while both CPTI and HSL are closely involved in the *β*-oxidation of fatty acids [47]. This result was consistent with those noted in the hemolymph TG and TC concentrations, which showed extremely low values in the control group. Based on these results, it can be speculated that niacin might stimulate the glucose uptake by hepatocytes from hemolymph in oriental river prawn assisted by GLUT2 [21]. Then, the increased glucose flux could up-regulate the glycolysis pathway, as consequently enhance glycogenesis, while suppress gluconeogenesis [48]. Meanwhile, the increased glucose uptake could enhance fatty acids synthesis, while depress the *β*-oxidation of fatty acids [49]. However, the processes mentioned above were all inhibited by the highest niacin doses (339.28 mg/kg), suggesting that excessive niacin intake might result in the disorders of glycolipid metabolism of oriental river prawn, as needs further validations.

## 5. Conclusion

Collectively, the present study demonstrated that dietary supplementation of niacin enhanced the growth performance and nutrients utilization of oriental river prawn. In addition, appropriate doses of niacin promoted the energy-sensing capability and glycolipid metabolism of this species. The optimum niacin demand of oriental river prawn was 168.01-169.08 mg/kg based on the regression analysis of weight gain and hepatopancreas niacin content.

## Figures and Tables

**Table 1:** Ingredients and proximate composition of the experimental diets.

Ingredient (%)	Experimental diets (supplemented niacin levels, mg/kg)					
	0	20	40	80	160	320
Fish meal	22.00	22.00	22.00	22.00	22.00	22.00
Casein	24.00	24.00	24.00	24.00	24.00	24.00
Gelatin	6.00	6.00	6.00	6.00	6.00	6.00
Fish oil	4.00	4.00	4.00	4.00	4.00	4.00
Soybean oil	2.00	2.00	2.00	2.00	2.00	2.00
Corn starch	25.00	25.00	25.00	25.00	25.00	25.00
Calcium dihydrogen phosphate	2.00	2.00	2.00	2.00	2.00	2.00
Premix^1^	1.00	1.00	1.00	1.00	1.00	1.00
Ecdysone	0.15	0.15	0.15	0.15	0.15	0.15
Carboxymethyl cellulose	3.00	3.00	3.00	3.00	3.00	3.00
Cholesterol	0.30	0.30	0.30	0.30	0.30	0.30
Lecithin	0.50	0.50	0.50	0.50	0.50	0.50
Ethoxyquin	0.50	0.50	0.50	0.50	0.50	0.50
Microcrystalline cellulose	9.55	9.55	9.55	9.55	9.55	9.55
Attractant (mg/kg)^2^	120.00	120.00	120.00	120.00	120.00	120.00
Niacin (mg/kg)^3^	0.00	20.00	40.00	80.00	160.00	320.00
Proximate composition (%)						
Dry matter	92.39	91.68	92.16	93.70	91.29	93.54
Crude protein	39.04	38.96	39.03	38.95	38.94	38.97
Crude lipid	17.15	17.11	16.96	16.92	17.16	17.09
Ash	6.94	6.96	6.95	6.99	7.01	6.97
Niacin (mg/kg)	15.75	37.62	56.62	97.78	176.32	339.28

^1^Premix supplied the following minerals and vitamins (per kg): CuSO_4_•5H_2_O, 2.0 g; FeSO_4_•7H_2_O, 25 g; ZnSO_4_•7H_2_O, 22 g; MnSO_4_•4H_2_O, 7 g; Na_2_SeO_3_, 0.04 g; KI, 0.026 g; CoCl_2_•6H_2_O, 0.1 g; vitamin A, 900,000 IU; vitamin D, 200,000 IU; vitamin E, 4,500 mg; vitamin K_3_, 220 mg; vitamin B_1_, 320 mg; vitamin B_2_, 1,090 mg; vitamin B_5_, 2,000 mg; vitamin B_6_, 500 mg; vitamin B_12_, 1.6 mg; vitamin C, 5,000 mg; pantothenate, 1,000 mg; folic acid, 165 mg; choline, 60,000 mg. ^2^ The attractant is betaine. ^3^ Purchased from Sigma Chemical Co., Ltd with a purity of more than 99%.

**Table 2:** Nucleotide sequences of the primers used to quantify gene expressions by real-time PCR.

Target genes	Primer sequence		Accession number or reference
*ampkα*	Forward (5′-3′)	TCACAGGGACAAAGGTAGCG	Sun et al., [50]
	Reverse (5′-3′)	TCTGCGAGCTTCCGATTCTT	

*sirt1*	Forward (5′-3′)	AGGCTCGAGAACCACTTTGG	MT505387.1
	Reverse (5′-3′)	GAAGTCCGACAAGCCACTCA	

*glut2*	Forward (5′-3′)	CTGACTGTGCCGTCGTACAT	CP062040.1
	Reverse (5′-3′)	ACACATAGGCCCACAGGTTG	

*hk*	Forward (5′-3′)	CATGGACAGCAGGATCTAC	KT932419
	Reverse (5′-3′)	AAACTGAACGTGAAGCCTAA	

*pk*	Forward (5′-3′)	TAAGGGACCTGAAATTCGTA	KP690140
	Reverse (5′-3′)	CCATCATCAACAAAAATCCT	

*gs*	Forward (5′-3′)	TTGAACCTTCGCGACCATGA	KP690145.1
	Reverse (5′-3′)	CTGGCCTGGAGTGGCAATAA	

*pc*	Forward (5′-3′)	CACCATTTGTAGCACACAAC	KP690141
	Reverse (5′-3′)	AACTCCATTTGCACCTCTAA	

*pepck*	Forward (5′-3′)	GGCATGGCGTAATGGTAGGA	JX435469.1
	Reverse (5′-3′)	ACCTTACCCTTGTGTTCCGC	

*g6p*	Forward (5′-3′)	CGTGGACCTTTCTTCATTAG	KP690144
	Reverse (5′-3′)	ACCATCAACCATTTGAGAAG	

*fas*	Forward (5′-3′)	CGTTCATGCTCGCGCAAATA	MK307767.1
	Reverse (5′-3′)	CGCCCACCTTAGTTCCAGTT	

*acc*	Forward (5′-3′)	CAAGGTCCACTACATGGTCT	KP690138
	Reverse (5′-3′)	ACTCTTCCCAAACTCTCTCC	

*cptI*	Forward (5′-3′)	AATTTTTGACTGGCTTCTCC	KP690136
	Reverse (5′-3′)	TCCATTCTGGAAATCATCTG	

*hsl*	Forward (5′-3′)	GAAGGCCAGCGCTAATTTCG	MK633965.1
	Reverse (5′-3′)	TCGAACCACCCATGAGAAGC	

*β-Actin*	Forward (5′-3′)	GTGCCCATCTACGAGGGTTA	FL589653.1
	Reverse (5′-3′)	CGTCAGGGAGCTCGTAAGAC	

*ampkα*: AMP-activated protein kinase *α*; *sirt1*: sirtuin 1; *glut2*: glucose transporter 2; *hk*: hexokinase; *pk*: pyruvate kinase; *gs*: glycogen synthase; *pc*: pyruvate carboxylase; *pepck*: phosphoenolpyruvate carboxykinase; *g6p*: glucose-6-phosphatase; *fas*: fatty acid synthase; *acc:* acetyl-CoA carboxylase; *cptI*: carnitine palmitoyltransferase I; *hsl*: hormone-sensitive lipase.

**Table 3:** Growth performance and feed utilization of oriental river prawn fed diets differing in niacin levels.

Dietary niacin levels (mg/kg)	IW (g)	FW (g)	SR (%)	WG (%)	SGR (%/day)	FI (g)	FCR	PER
15.75	0.17 ± 0.01	0.55 ± 0.02^a^	70.50 ± 4.08	81.61 ± 12.93^a^	0.84 ± 0.10^a^	1.16 ± 0.18^a^	4.30 ± 0.35^b^	1.16 ± 0.17^a^
37.62	0.18 ± 0.01	0.57 ± 0.02^a^	83.53 ± 4.93	117.83 ± 11.73^ab^	1.21 ± 0.12^ab^	2.00 ± 0.26^ab^	3.40 ± 0.23^ab^	2.00 ± 0.26^ab^
56.62	0.19 ± 0.01	0.62 ± 0.01^ab^	82.38 ± 3.44	134.97 ± 8.99^ab^	1.11 ± 0.07^ab^	1.69 ± 0.16^ab^	3.53 ± 0.16^ab^	1.69 ± 0.16^ab^
97.78	0.16 ± 0.01	0.61 ± 0.02^ab^	85.50 ± 3.39	145.92 ± 8.33^ab^	1.23 ± 0.08^ab^	1.98 ± 0.15^ab^	3.32 ± 0.10^a^	1.98 ± 0.15^ab^
176.32	0.17 ± 0.01	0.70 ± 0.03^b^	88.62 ± 2.13	179.93 ± 23.77^b^	1.46 ± 0.12^b^	2.47 ± 0.36^b^	3.11 ± 0.15^a^	2.47 ± 0.36^b^
339.28	0.20 ± 0.01	0.64 ± 0.04^ab^	81.75 ± 7.25	138.19 ± 18.38^ab^	1.27 ± 0.11^ab^	2.17 ± 0.28^ab^	3.30 ± 0.15^a^	2.17 ± 0.28^ab^
Polynomial contrasts								
Linear	ns	*P* < 0.001	ns	*P* < 0.05	*P* < 0.05	*P* < 0.05	*P* < 0.05	*P* < 0.05
Quadratic	ns	ns	ns	ns	ns	ns	*P* < 0.05	ns
Cubic	ns	ns	ns	ns	ns	ns	ns	ns

IW: initial weight; FW: final weight; SR: survival rate; WG: weight gain; SGR: specific growth rate; FI: feed intake; FCR: feed conversion ratio; PER: protein efficiency ratio. Data are represented as means ± S.E.M (*n* =4). Values in the same column with different superscripts are significantly different (*P* < 0.05). ns; not significant.

**Table 4:** Hepatopancreas niacin contents and hemolymph metabolites concentrations of oriental river prawn fed diets differing in niacin levels.

Dietary niacin levels (mg/kg)	Hepatopancreas niacin contents (mg/kg)	GLU (mmol/L)	TP (mmol/L)	TG (mmol/L)	TC (mmol/L)
15.75	60.28 ± 0.27^a^	82.21 ± 3.04^ab^	0.71 ± 0.08^a^	0.16 ± 0.02^ab^	0.06 ± 0.01^ab^
37.62	63.11 ± 0.05^b^	87.58 ± 1.80^b^	0.72 ± 0.02^ab^	0.47 ± 0.10^b^	0.09 ± 0.01^c^
56.62	66.50 ± 0.43^c^	79.54 ± 4.06^ab^	0.96 ± 0.01^ab^	0.10 ± 0.05^a^	0.08 ± 0.01^bc^
97.78	68.00 ± 0.30^c^	79.65 ± 2.29^ab^	0.89 ± 0.06^ab^	0.12 ± 0.06^ab^	0.05 ± 0.01^ab^
176.32	73.00 ± 0.58^d^	76.37 ± 1.88^a^	1.00 ± 0.04^b^	0.39 ± 0.27^ab^	0.04 ± 0.01^a^
339.28	74.10 ± 0.47^d^	76.11 ± 1.42^a^	0.96 ± 0.10^ab^	0.090 ± 0.02^a^	0.05 ± 0.01^ab^
Polynomial contrasts					
Linear	*P* < 0.001	*P* < 0.05	*P* < 0.05	*P* < 0.05	*P* < 0.05
Quadratic	ns	ns	ns	ns	ns
Cubic	ns	ns	ns	ns	*P* < 0.05

GLU: glucose; TP: total protein; TG: triglyceride; TC: total cholesterol. Data are represented as means ± S.E.M (*n* =4). Values in the same column with different superscripts are significantly different (*P* < 0.05). ns: not significant.

## Data Availability

The data sets generated and/or analyzed during the current study are available from the corresponding author on request.
